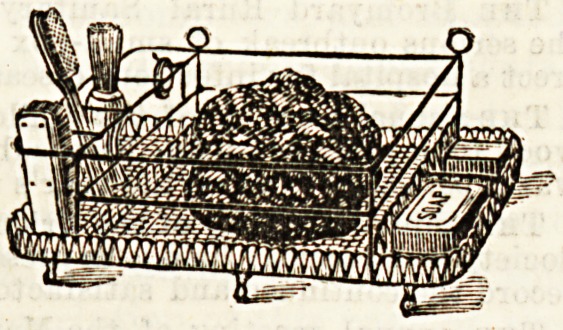# Practical Departments

**Published:** 1894-02-17

**Authors:** 


### PRACTICAL DEPARTMENTS.

### TOILET TRAYS.

In THE HOSPITAL for July 8th, 1893, we gave a description of a china toilet tray, specially designed for use in the sick room, by Mr. W. P. Brook. On critically examining this very useful invention we suggested that it would be of still more service if made in unbreakable ware. We are glad to be able to show that this suggestion has been followed by the inventor, and the present illustration is of a white enamelled iron tray. The idea is one to be recommended, and these little trays will be found really time-saving and useful in carrying toilet necessaries, soap and sponges, to the bedside of the patient. In nurseries, too, they will be helpful by keeping all the small etceteras together for the baths, their unbreakable character making it safe for them to be left on the floor or chair, even when nurse's back is turned, without fear of a smash ensuing.

Mr. Brook has further elaborated his idea by having it carried out in the form of a wire tray, for use in bath-room or lavatory, with divisions, as shown in the accompanying illustration, for holding sponges, soap, tooth and nail brushes, &c. It is really a dainty-looking contrivance, and both trays are to be commended for being thoroughly sanitary in character and easily cleanable. The wire tray, as well as the other, is provided with a special corner for rings.

The trays may be ordered from any ironmonger's or brush manufacturer's, and our readers will do well to give Mr. Brook's convenient invention a trial. We feel sure it has only to be known to be appreciated.

### BERKEFELD FILTER.

This new form of filter has been introduced by the Berkefeld Filter Company,, 121, Oxford Street. The system upon which it is constructed is that of the abstraction of bacteria, or any other visible, so to speak, atomic matter from water by passing it under pressure through very fine porous baked china or earthenware, made up of substances claimed (from a certain crystalline formation) to offer such small orifices that nothing but water can pass through. This substance, Kieselquhr (Fossil Earth) is composed of the silicious skeletons of diatomaceæ, their peculiar formation giving an enormous number of exceedingly minute pores, thus affording a free passage for the liquid, and at the same time stopping that of the minutest suspended organic or inorganic matter, while their hard silicious nature gives a firm and practically indestructible material. The filtration is accomplished by means of cylinders, which give a satisfactory flow of liquid, but prevent the entry of the most minute animalculæ. The harmful deposits can be easily brushed away.

On careful examination and testing we find that the Berkefeld filter is likely to prove of more value in the laboratory, for the preparation of liquids for experimental purposes, than for the purifying of water for drinking. The tests we have caused to be made prove that, in the practical working, whilst insoluble matter is undoubtedly all arrested in its pores, soluble impurities pass through unaltered. This latter is the most dangerous form of impurity to be dealt with in water, and for ordinary purposes we cannot, therefore, recommend the Berkefeld filter, its disadvantages being: 1. That water can only be passed through under pressure, which is not always attainable. 2. Soluble impurities are not oxydised or eliminated, or in any way altered or affected during the process of filtration. Where used for filtering drinking water, therefore, great care should be observed in the cleansing of the cylinders, which may be thoroughly sterilised by boiling, and the water should afterwards be passed through an ordinary carbon filter. But, as we have said, for experimental purposes the Berkefeld filter will be found very useful.

## Figures and Tables

**Figure f1:**
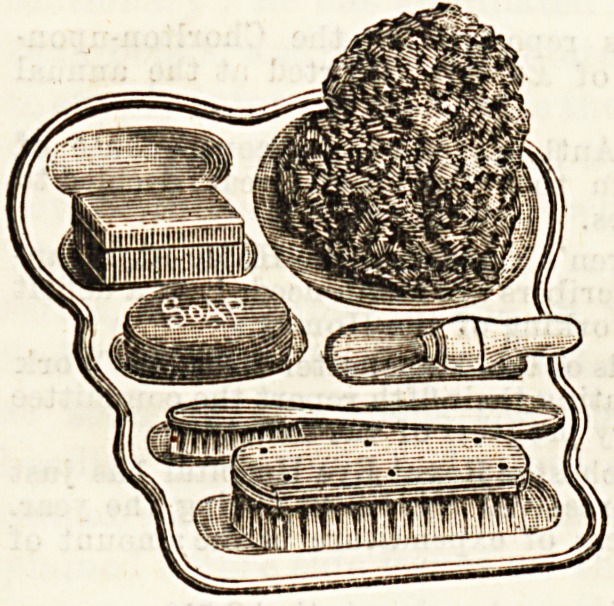


**Figure f2:**